# Awareness of First-Aid Management of Epistaxis Among Individuals in Al-Ahsa Region

**DOI:** 10.7759/cureus.54625

**Published:** 2024-02-21

**Authors:** Hassan A Alkhalaf, Shoug T Alshoug, Abdul Qadeer Memon

**Affiliations:** 1 Surgery Department, King Faisal University, Al Ahsa, SAU; 2 Radiology Department, King Faisal University, Al Ahsa, SAU

**Keywords:** bleeding, ent, first aid, epistaxis, awareness

## Abstract

Introduction

Epistaxis is among the most common emergencies in the Ear, Nose, and Throat department. The vast majority of these patients are treated with basic first-aid management. Our study aims to assess the awareness of first‑aid management of epistaxis among individuals in the Al-Ahsa region, Kingdom of Saudi Arabia.

Materials and methods

This is a cross-sectional study conducted among the male and female population in Al-Ahsa, Saudi Arabia, during March 2023, and it included all participants available at the time of the study. All the data were obtained using an online questionnaire.

Results

The study included 385 participants; 213 (55.3%) were females and 172 (44.7%) were males. Based on the correlation between our variables, 235 (61%) of the participants had a good awareness score while only 150 (39%) had a poor score. Our findings show that 113 (65.7%) of males had a good level of awareness and 122 (57.3%) of females had a good level of awareness regarding epistaxis. Participants who had gone through an experience of the treatment of any patients with epistaxis had better awareness about first-aid management of epistaxis than participants who had never gone through the experience.

Conclusion

The knowledge and awareness regarding first-aid management of epistaxis among the general population was satisfactory. However, we should increase the level of knowledge of individuals who did not treat any people with epistaxis because there are many cases of epistaxis in Al-Ahsa However, more integrated educational materials should be available to the general population to improve their overall knowledge. Social campaigns in public areas will enhance the level of knowledge regarding epistaxis management among the general population.

## Introduction

Epistaxis, generally referred to as "bleeding from the nose," continues to be one of the most widely recognized Ear, Nose, and Throat (ENT) crises exhibited in Emergency Departments (EDs) around the world. In the United States (US), around 1.7% of all ED visits are because of epistaxis. What's more, around 1 out of 200 ED visits in the United States (US) are because of epistaxis [1]. The bleeding can be minor and stop unexpectedly or so extreme as to be deadly [2]. For most, epistaxis happens from the front portion of the nasal pit, mainly from Kiesselbach's plexus [3]. Epistaxis most likely occurs even more often during the dry, cold winter. It is thought to happen more routinely in males than females, and there is an expanding repeat with age [4].

Nasal bleeding can be a result of either systemic or local causes. Systemic factors involve coagulopathy, blood diseases, anticoagulant consumption, and arterial high blood pressure. In contrast, local factors include upper airway infections, nasal allergies, foreign body introduction into the nasal cavity, trauma, and septal perforation [5]. Most patients who are affected with epistaxis can settle with standard first aid. On the other hand, a few epistaxis episodes require hospital admission [6]. Even though epistaxis is common, first aid measures with proper understanding are necessary to handle epistaxis without medical facilities [6-8]. Some reports additionally indicated poor information on the emergency treatment of epistaxis in the general population and among health experts [9]. Epistaxis is more common in nonhospital settings, and most situations are managed with modest first-aid interventions. Nonmedical persons can implement these steps at the scene of the event until recovery or professional medical treatment is found. As a result, nonmedical persons (such as teachers, parents, and colleagues) need to learn epistaxis first-aid techniques. Therefore, we aimed to assess the awareness of first‑aid management of epistaxis among Individuals in the Al-Ahsa region.

## Materials and methods

Study design and setting

A cross-sectional study was conducted among the male and female populations in Al-Ahsa, Saudi Arabia, in March 2023. It included all participants available at the time of the study. All data were obtained using an online questionnaire. Three hundred eighty-five Saudi and non-Saudi nationals were included in the study, provided they agreed to participate and were above 18. The exclusion criteria included any participants below 18 years old. The purpose of the research was explained to the participants, and informed consent was obtained before the start of the study. 

Participants and data collection 

The questionnaire was adapted from a previous study, which consisted of two parts [10]. The first part contained questions about the respondents' demographic data (i.e., age, nationality, gender, and educational level). The second part included nine true/false questions regarding the information and knowledge about epistaxis. The Institutional Review Board at King Faisal University authorized this research with research No. KFU-REC-2024-JAN-ETHICS1950

Data analysis

The data were collected, reviewed, and then fed to Statistical Package for Social Sciences version 21 (IBM Corp., Armonk, NY, USA). All statistical methods were two-tailed, with an alpha level of 0.05, considering significance if the P value is less than or equal to 0.05. Overall knowledge level regarding epistaxis first aid was assessed by summing up discrete scores for different correct knowledge items. The overall knowledge score was categorized as a poor level if the participant's score was less than 60%, and a good level of knowledge was considered if the participants' score was 60% or more of the overall score. Descriptive analysis was done by prescribing frequency, distribution, and percentage for study variables, including participants' personal data, education, history of epistaxis, and training courses. Also, knowledge regarding epistaxis first aid was tabulated while the overall knowledge was graphed. Cross-tabulation for showing factors associated with public knowledge of epistaxis first aid was carried out with Pearson's chi-square test for significance and the exact probability test if there were small frequency distributions.

## Results

A total of 385 participants completed the study questionnaire. Participants ages ranged from 18 to more than 50 years, with a mean age of 27.6 ± 11.9 years. Three hundred sixty-seven (367; 95.3%) were Saudi and 213 (55.3%) were females. As for education level, 214 (55.6%) were university graduates and 116 (30.1%) had a secondary level of education. A total of 194 (50.4%) took first aid training, 176 (45.7%) previously had epistaxis, and 192 (49.9%) treated someone with epistaxis (Table 1).

**Table 1:** Personal characteristics of the participants, Al-Ahsa, Saudi Arabia

Age in years	No.	%
18-25	191	49.6%
26-35	129	33.5%
36-50	49	12.7%
> 50	16	4.2%
Nationality		
Saudi	367	95.3%
Non-Saudi	18	4.7%
Gender		
Male	172	44.7%
Female	213	55.3%
Educational level		
Below secondary	55	14.3%
Secondary	116	30.1%
University / above	214	55.6%
Have you taken any first aid course?		
Yes	194	50.4%
No	191	49.6%
Have you had epistaxis?		
Yes	176	45.7%
No	209	54.3%
Have you treated anyone with epistaxis?		
Yes	192	49.9%
No	193	50.1%

Table 2 shows public knowledge and awareness regarding epistaxis first aid in Al-Ahsa, Saudi Arabia. Regarding risk factors knowledge, 318 (82.6%) said that exposure to hot places and hot weather exacerbate epistaxis, 317 (82.3%) reported that doing strenuous exercises exacerbates epistaxis, 301 (78.2%) reported that hot baths exacerbate epistaxis, 268 (69.6%) agreed that hot beverages aggravate epistaxis, and 268 (69.6%) know that smoking causes epistaxis to recur. Considering epistaxis first aid and management, 333 (86.5%) know that they should stop it or control it by pressure, 281 (73%) told that they should try to stop the bleeding by applying ice on nose, head, or between the eyes, 219 (56.9%) know that during epistaxis, they should tilt their head forward, 203 (52.7%) know that they should go to ER if bleeding lasts for 20 minutes, 175 (45.5%) know that they should press upper part of the nose, and 170(44.2%) know that they should press on nose for 5-10 minutes.

**Table 2:** Public knowledge and awareness regarding epistaxis first aid in Al-Ahsa, Saudi Arabia

Domain	Items		No.	%
Risk factors	Hot beverages exacerbate epistaxis	True statement	268	69.6%
		False statement	117	30.4%
	Hot baths exacerbate epistaxis	True statement	301	78.2%
		False statement	84	21.8%
	Exposure to hot places and hot weather exacerbate epistaxis	True statement	318	82.6%
		False statement	67	17.4%
	Doing strenuous exercises exacerbates epistaxis	True statement	317	82.3%
		False statement	68	17.7%
	Sneezing is related to epistaxis	True statement	247	64.2%
		False statement	138	35.8%
	Moisturizing the nose reduces epistaxis	True statement	291	75.6%
		False statement	94	24.4%
	Smoking causes epistaxis to recur	True statement	268	69.6%
		False statement	117	30.4%
Management methods	I will try to stop the bleeding by blocking the nose with tissue paper, cotton, or any similar object	True statement	236	61.3%
		False statement	149	38.7%
	I will try to stop the bleeding by applying ice on nose, head, or between eyes	True statement	281	73.0%
		False statement	104	27.0%
	If I have epistaxis	I will try to stop it or control it by pressure	333	86.5%
		I will leave it	52	13.5%
	Site of applied pressure	Upper part of the nose	175	45.5%
		Bottom of the nose	148	38.4%
		I do not know	62	16.1%
	How long did you press on the area?	< 5 minutes	175	45.5%
		5-10 minutes	170	44.2%
		11-20 minutes	33	8.6%
		> 20 minutes	7	1.8%
	I will change the position of my head by:	Tilting my head forward	219	56.9%
		Tilting my head backward	166	43.1%
	When is the right time to go to the emergency room?	After 20 minutes	203	52.7%
		After 40 minutes	97	25.2%
		After 60 minutes	32	8.3%
		At any time	53	13.8%
	How much bleeding is considered a large amount?	> one cup	68	17.7%
		One‑full cup	190	49.4%
		One‑half cup	62	16.1%
		One‑third cup	16	4.2%
		One‑quarter cup	49	12.7%

**Figure 1:**
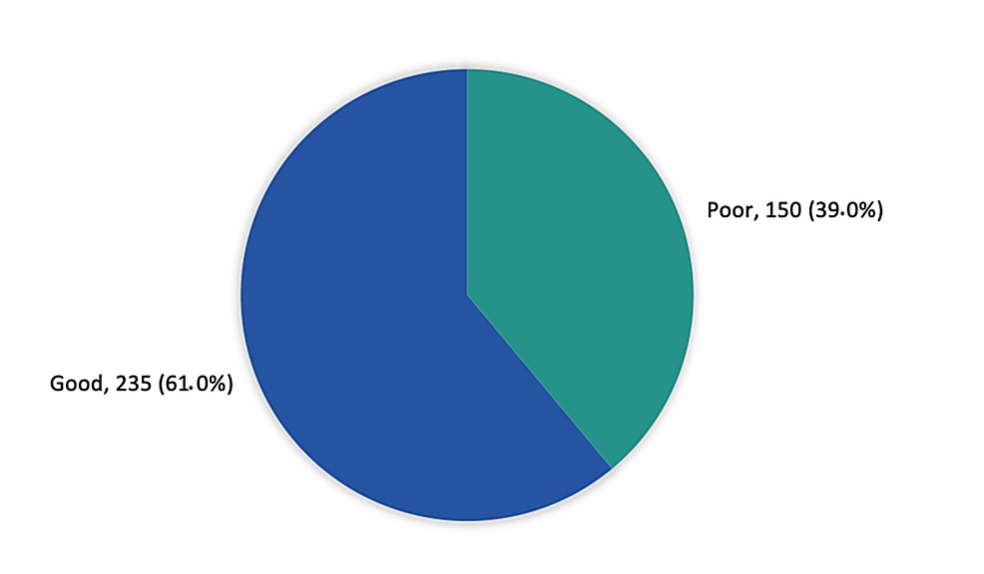
Overall public knowledge and awareness regarding epistaxis first aid in Al-Ahsa, Saudi Arabia

Figure 1 shows the overall public knowledge and awareness regarding epistaxis first aid in Al-Ahsa, Saudi Arabia. Exact 235 (61%) of the study participants had a generally good knowledge and awareness regarding epistaxis first aid while 150 (39%) had a poor knowledge level.

Table 3 lists factors associated with participant's knowledge regarding epistaxis first aid. One hundred one (101; 78.3%) of participants aged 26-35 had an overall good knowledge level versus 48.7% of others aged 15-25 with recorded statistical significance (P=0.001). Also, 41 (74.5%) of participants with a lower level of education had an overall good knowledge level compared to 117 (54.7%) of others with a university level of education (P=0.010). A good knowledge level regarding epistaxis first aid was detected among 145 (74.7%) of those who had first-aid training courses compared to 90 (47.1%) of others who did not (P=0.001). Likewise, 141 (80.1%) of participants who previously had epistaxis had a good knowledge of first aid compared to 94 (45%) of others (P=0.001). Similarly, good knowledge was detected among 152 (79.2%) of participants who treated anyone with epistaxis versus 83 (43%) of others who did not (P=0.001).

**Table 3:** Factors associated with participants' knowledge of epistaxis first * P < 0.05 (significant)

Factors	Overall knowledge level				p-value
	Poor		Good		
	No.	%	No.	%	
Age in years					0.001*
18-25	98	51.3%	93	48.7%	
26-35	28	21.7%	101	78.3%	
36-50	18	36.7%	31	63.3%	
> 50	6	37.5%	10	62.5%	
Nationality					0.616
Saudi	144	39.2%	223	60.8%	
Non-Saudi	6	33.3%	12	66.7%	
Gender					0.092
Male	59	34.3%	113	65.7%	
Female	91	42.7%	122	57.3%	
Educational level					0.010*
Below secondary	14	25.5%	41	74.5%	
Secondary	39	33.6%	77	66.4%	
University/above	97	45.3%	117	54.7%	
Have you taken any first aid course?					0.001*
Yes	49	25.3%	145	74.7%	
No	101	52.9%	90	47.1%	
Have you had epistaxis?					0.001*
Yes	35	19.9%	141	80.1%	
No	115	55.0%	94	45.0%	
Have you treated anyone with epistaxis?					0.001*
Yes	40	20.8%	152	79.2%	
No	110	57.0%	83	43.0%	

## Discussion

Our study aimed to measure individuals' understanding of first-aid management of epistaxis in the Al-Ahsa region because it is common in this region and leads to a huge amount of bleeding. Epistaxis is one of the most common ENT emergencies, affecting around 1.7% of all ED visits. Furthermore, epistaxis accounts for approximately one out of every 200 ED visits in the US [1]. In our current study, 61% of the participants had good awareness and 39% had poor awareness. In addition, this was one of the few research studies completed in Saudi Arabia, demonstrating the need for additional studies in the field. According to our findings, males had a greater level of awareness than females. In similar research on the level of knowledge about handling epistaxis, just 11.3% of the medical staff at Kenya's Accident and ED were aware of how to manage epistaxis correctly [9]. In another study among health professionals, <40% of participants knew how to correctly apply pressure to the nose [11]. Approximately 63.2% of the population of Saudi Arabia had poor awareness regarding first‐aid management for epistaxis [10].

In our study, 86.5% of the participants answered, "If I have epistaxis, I will try to stop it or control it by pressure." In another study, 81.4% answered, "I will try to stop it or control it by pressure" [12]. In addition, 56.9% of our participants answered that "they will tilt their head forward during the event of epistaxis." However, another study shows that 65% of participants responded that "they will tilt their head backward during the event of epistaxis" [12]. In contrast, a similar study in Saudi Arabia used the same questionnaire as ours and reported that only 15% of the population selected lowering their heads backward as part of epistaxis management [10].

In our study, 50.4% of the participants took a first-aid course on the management of epistaxis. However, according to another study at the Accident and ED of Kenyatta National Hospital, over three-quarters (75.7%) of the participants had no formal training in first aid treating epistaxis [9]. In 2008, Ho EC et al. found a similarly high proportion (83.3%) of accident and emergency workers who had not received a formal education in the first-aid management of epistaxis [13]. This limitation of training may lead to a poor understanding of first-aid measures, as it is predicted that training has a significant influence on knowledge of the measures.

In our study, 61.3% of responders attempted to stop the bleeding by plugging the nose with tissue paper, cotton, or other similar material. Another research with similar questions found that 74.4% of the participants reported they would try to stop the bleeding by plugging the nose with tissue paper, cotton, or any similar material [12]. Adhikari et al. (2006) discovered that nasal packing was the most employed first-line measure among accident and emergency clinical professionals [14].

In general, the attitude of our participants towards first aid in epistaxis was good. In addition, our participants who had treated patients with epistaxis had better awareness of first-aid management than those who had never treated patients with epistaxis. This will tell us that we need to increase the awareness of first-aid management of epistaxis, especially in the school age by doing social campaigns and visiting schools, which could be deadly if not managed properly.

This study's strength is that it's one of the first studies carried out in the Al-Ahsa Region to assess the attitude of first-aid management of epistaxis. The limitation of our study is that since the study was conducted among a broad community, it is not reasonable to generalize the study's findings to the entire Al-Ahsa population. Given the lack of research in this sector, this may be a topic for future studies. Also, only a small number of participants were above 50 years old, and we should measure their knowledge of first-aid management of epistaxis.

## Conclusions

Knowledge and awareness regarding the first‐aid management of epistaxis among the general population were satisfactory. However, more integrated educational materials should be available to the general population to improve their overall knowledge. Social campaigns in public areas will enhance the level of knowledge regarding epistaxis management among the general population.
